# Identification of Stroke and TIA in Patients With Acute Dizziness, Vertigo or Imbalance in Emergency Departments of Primary Care Hospitals: Early Experiences With a Network-Based Telemedical Approach

**DOI:** 10.3389/fneur.2022.766685

**Published:** 2022-03-02

**Authors:** Peter Müller-Barna, Christina Leinweber, Julia Pfaffenrath, Nina Schütt-Becker, Rascha von Martial, Susanne Greck, Nikolai Hubert, Holger Rambold, Roman Haberl, Gordian Jan Hubert

**Affiliations:** ^1^Department of Neurology, TEMPiS Telestroke Center, München Klinik, Academic Teaching Hospital of the Ludwig-Maximilians-University, Munich, Germany; ^2^Department of Neurology, InnKlinikum Altötting, Altötting, Germany; ^3^Department of Neurology, University of Regensburg, Regensburg, Germany; ^4^MVZ Kliniken Mühldorf, Mühldorf am Inn, Germany

**Keywords:** dizziness, vertigo, telemedicine, emergency medicine, stroke, diagnostic method, acute vestibular syndrome

## Abstract

**Background:**

Acute dizziness, vertigo, and imbalance are frequent and difficult to interpret symptoms in the emergency department (ED). Primary care hospitals often lack the expertise to identify stroke or TIA as underlying causes. A telemedical approach based on telestroke networks may offer adequate diagnostics and treatment.

**Aim:**

The aim of this study is to evaluate the accuracy of a novel ED algorithm in differentiating between peripheral and central vestibular causes.

**Methods:**

Within the Telemedical Project for Integrative Stroke Care (TEMPiS), a telemedical application including a videooculography (VOG) system was introduced in 2018 in 19 primary care spoke hospitals. An ED triage algorithm was established for all patients with acute dizziness, vertigo, or imbalance of unknown cause (ADVIUC) as a leading complaint. In three predefined months, all ADVIUC cases were prospectively registered and discharge letters analyzed. Accuracy of the ED triage algorithm in differentiation between central and peripheral vestibular cases was analyzed by comparison of ED diagnoses to final discharge diagnoses. The rate of missed strokes was calculated in relation to all cases with a suitable brain imaging. Acceptance of teleconsultants and physicians in spoke hospitals was assessed by surveys.

**Results:**

A total number of 388 ADVIUC cases were collected, with a median of 12 cases per months and hospital (IQR 8–14.5). The most frequent hospital discharge diagnoses are vestibular neuritis (22%), stroke/TIA (18%), benign paroxysmal positioning vertigo (18%), and dizziness due to internal medicine causes (15%). Detection of a central vestibular cause by the ED triage algorithm has a high sensitivity (98.6%), albeit poor specificity (45.9%). One stroke out of 32 verified by brain scan was missed (3.1%). User satisfaction, helpfulness of the project, improvement of care, personal competence, and satisfaction about handling of the VOG systems were rated consistently positive.

**Discussion:**

The concept shows good acceptance for a telemedical and network-based approach to manage ADVIUC cases in the ED of primary care hospitals. Identification of stroke cases is accurate, while specificity needs further improvement. The concept could be a major step toward a broadly available state of the art diagnostics and therapy for patients with ADVIUC in primary care hospitals.

## Introduction

Acute dizziness and vertigo are frequent and difficult to diagnose symptoms in the emergency department (ED) (1–3). Since stroke is the underlying cause in about 5% of these cases (4, 5) but missed in about 35% of stroke cases, especially when symptoms are mild and transient (6–9), a specific concept is required. Therefore, a workflow in the ED is desirable which identifies all stroke cases correctly for rapid admission to a stroke unit and applies immediate therapy such as intravenous thrombolysis if indicated. The most promising approach seems to use a battery of different oculomotor tests like the HINTS (head impulse – nystagmus – test of skew) or the HINTS plus exam (10–14), which identify strokes based on clinical examinations better than an initial MRI (9, 12). Adding the video head impulse test (vHIT) to quantify the vestibular ocular reflex further improves the effectiveness of the algorithm (15–17).

As those tests are applied by trained neurootologists, our challenge is to bring the necessary expertise to the bedside in ED of primary care hospitals, which mostly do not employ a neurologist in the ED. Our favored solution is telemedicine, as is already used with success in treating stroke, known as telestroke (18, 19). A telemedical consultation program for acutely dizzy patients bringing neurootological expertise in the ED of the same hospital reported higher diagnostic accuracy and lower employment of computed tomography scans (20). The employment of smartphones for vHIT measurement may facilitate this development in the future (21). A clinical trial comparing video-oculography (VOG) guided care to standard care in EDs is ongoing (Acute Video-Oculography for Vertigo in Emergency Rooms for Rapid Triage [AVERT] trial) (22).

Current telestroke networks seem ideal for the additional implementation of telemedical care for acutely dizzy patients, as there are close synergies to be expected (medically – because of the underlying etiology of stroke – and technically – because of the similar infrastructure needed). The logistics developed for telestroke could be extended for dizzy patients, regarding teleconsultation workflow, specific treatment standards, training, and quality management. Additional resources needed are mainly one expert neurootologist and one vestibular rehabilitation therapist in the network center, VOG systems in all spokes and sufficient resources for training and quality management (23).

This study evaluates our telematic approach to diagnose acute dizziness, vertigo, and imbalance in our telemedical stroke network TEMPiS in Bavaria, Germany, with the main focus on the diagnostic accuracy of the ED algorithm in differentiating between peripheral and central vestibular diseases. Results of the remote VOG examination including telemedical HINTS and vHIT are reported in another paper (24).

## Materials and Methods

**Figure 1:**
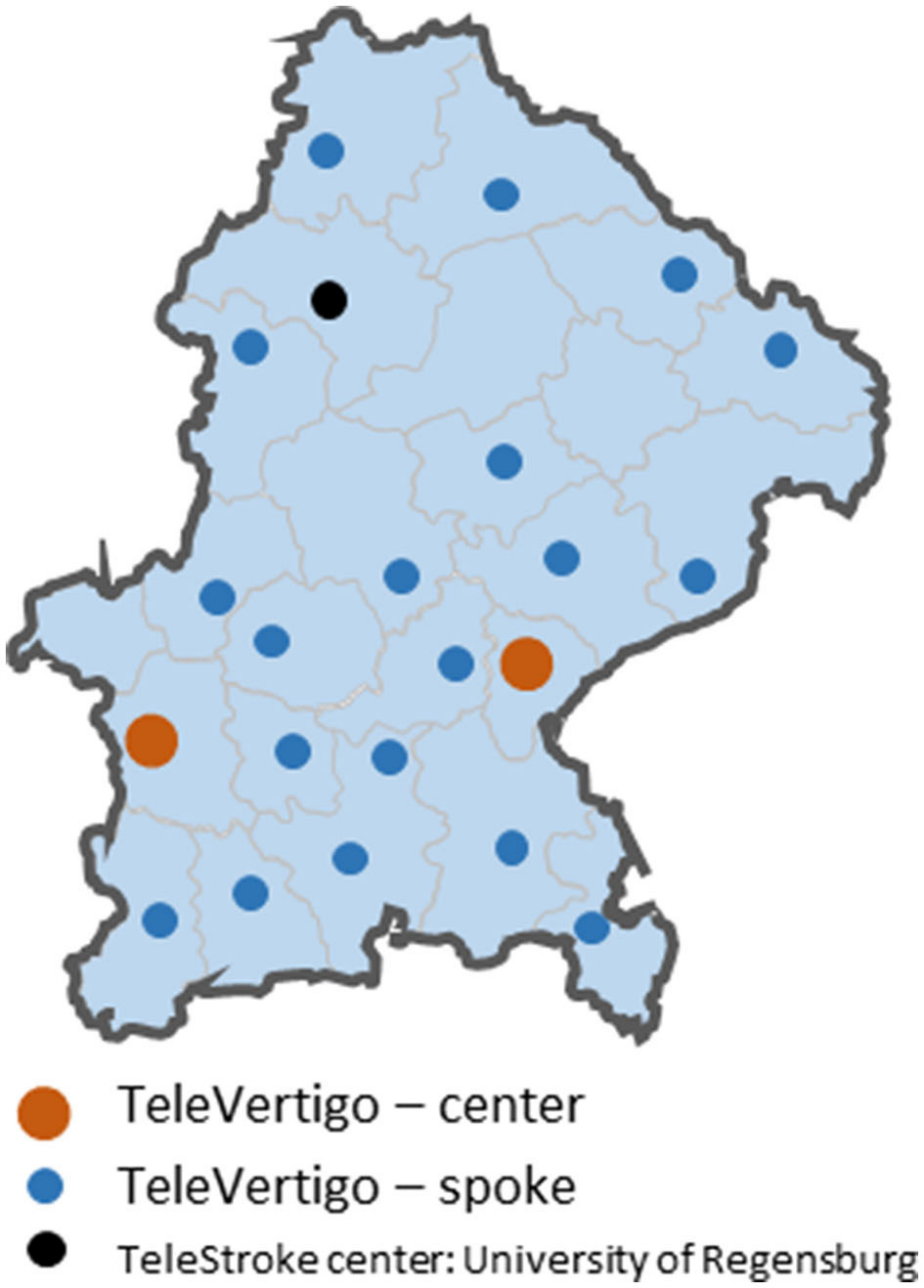
Map of the southeastern part of Bavaria, Germany with all TeleVertigo hospitals.

As telestroke is already implemented in our region of South-East Bavaria, we built our concept on this telematic system. The Telemedical Project for Integrative Stroke Care (TEMPiS) is a stroke network consisting of two hubs and 24 spokes and was implemented in 2003 (18, 19). A total of 7,427 and 7,337 teleconsultations were performed in 2019 and 2020, respectively. TeleVertigo was introduced in 2018 in 19 spoke hospitals as an add-on to the preexisting telestroke collaboration (Figure 1). The detailed concept was already published (23). The catchment area of the 19 hospitals sums up to a population of 1,987,414 inhabitants (25). Six of these spokes have a neurology department on-site with 24/7 availability of a neurologist.

**Figure 2:**
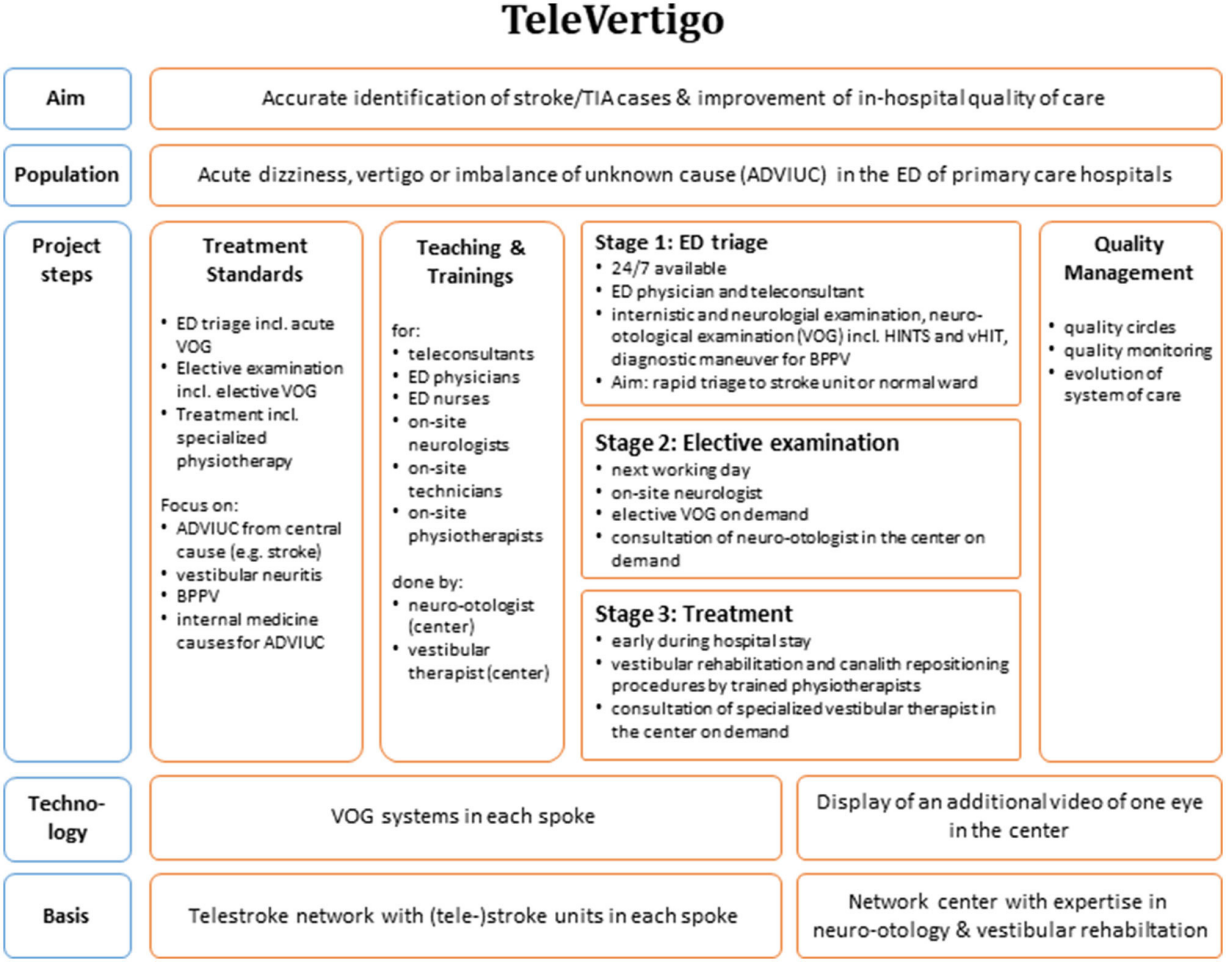
Overview of the major components of the TeleVertigo project.

**Figure 3:**
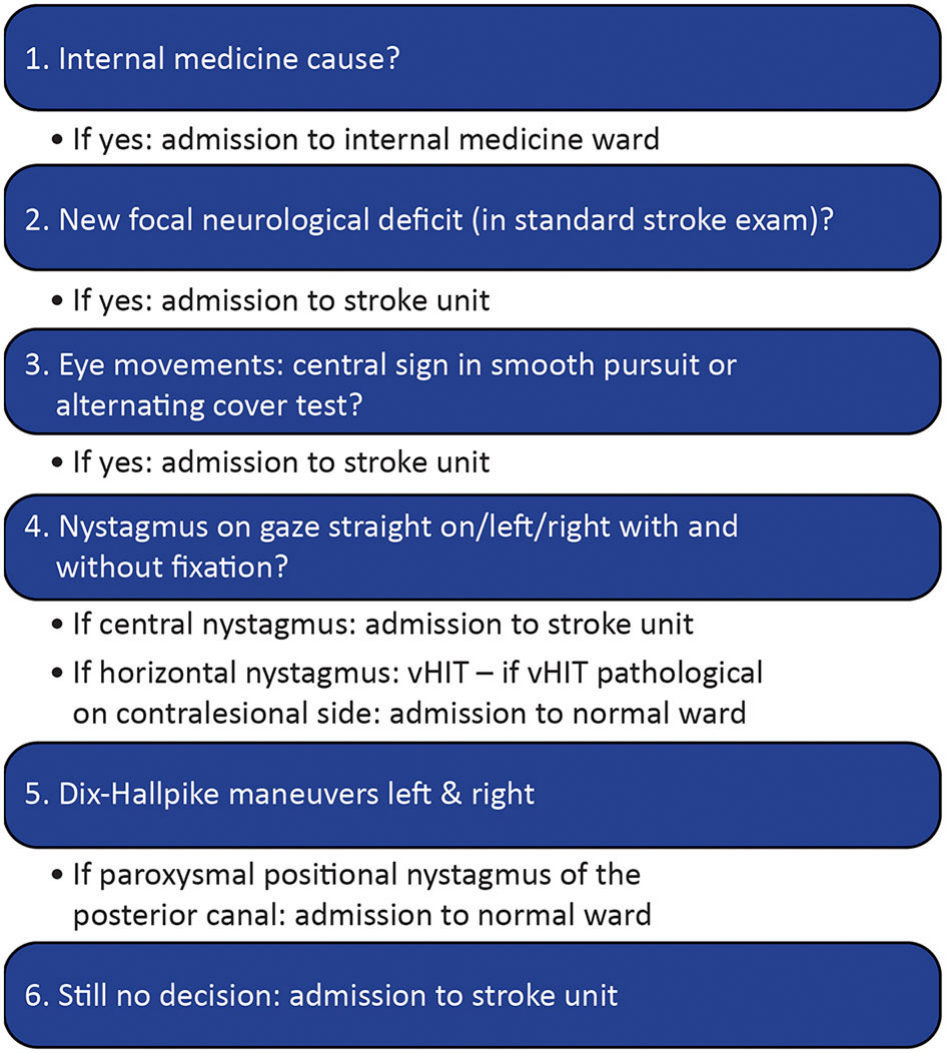
ED triage algorithm for all cases of acute dizziness, vertigo, or imbalance.

In brief, our innovative telemedical concept addresses all patients who were admitted to an ED of the participating primary care hospitals due to acute dizziness, vertigo, or imbalance of unknown cause (ADVIUC) as a leading complaint (Figure 2). The key technical diagnostic tool is a VOG system embedded in a standard videoconferencing system. In contrast to standard videoconferencing systems (26), the added VOG system enables vestibular testing by telemedicine. The concept comprises three stages: (1) telemedical triage in the ED (including vHIT and fast track VOG; Figure 3) for reliable and rapid decision-making on acute treatment, including stroke treatment if indicated: This triage uses a telemedical examination of oculomotor signs, the vHIT and the diagnostic Dix-Hallpike-maneuvers for benign paroxysmal positional vertigo (BPPV) of the posterior semicircular canals. For this purpose, a VOG system (“video goggles”; ICS Impulse Type 1085 in combination with the OTOsuite Vestibular software, version 4.10 Build 1341, Natus Medical Denmark ApS, Taastrup, Denmark) was included in the videoconferencing system in all participating hospitals; (2) elective clinical examination including quantitative VOG and finding of the causal diagnosis: In cases of ADVIUC, an elective neurological examination by the vascular neurologist is done next day. Vascular neurologists are thoroughly trained within this project in diagnosing dizziness and vertigo. Additionally, a VOG including nystagmus detection, tests for skew deviation, visually guided saccades, smooth pursuit eye movements, vHIT, and Dix-Hallpike-maneuvers is performed by trained technicians or physical therapists. On demand, a neurootologist of the network center evaluates this VOG remotely and supports spoke physicians in diagnosis and therapy. (3) Adequate treatment including vestibular rehabilitation and canalith repositioning procedures: Physiotherapy was established for all patients with ongoing dizziness, vertigo, or imbalance, including canalith repositioning maneuvers and vestibular rehabilitation. To obtain high standards, a physical therapist of the network center specialized in vestibular disorders offers regular training to the physical therapists in the spoke hospitals.

In 2018, we developed project standards for acute and elective diagnosis and treatment of patients with dizziness, vertigo, and imbalance, and also specific diagnostic and treatment standards for benign paroxysmal positional vertigo, vestibular neuritis, and dizziness of central origin, based on the criteria of the Bárány Society (27–29). We trained 180 physicians, therapists, and technicians of the participating hospitals in these standards in 15 sessions lasting 6 h each, consisting of 3.5 h of theoretical training and 2.5 h of practical training. An exit test or any other credential was not collected.

### Study Protocol

For project evaluation, evaluation periods with full documentation of all admitted patients in the EDs of the participating hospitals were predefined. In compliance with the capacity of participating primary care hospitals, we tailored the evaluation effort to 3 months (11/2019, 3/2020, and 10/2020). A screening registry was designed, and all participating hospitals were asked to register all patients with the leading symptom of acute dizziness, vertigo, or imbalance of unknown cause (ADVIUC) in screening lists. “Unknown cause” was defined as follows: (1) In the ED, no history of episodic vertigo or dizziness with the same quality of symptoms like in the actual presentation was reported, and (2) in the first short triage done by ED physicians, an underlying reason for the acute dizziness symptom is not obvious. This triage includes a short internal medicine and neurological examination and also the assessment of vital parameters (heart rate, blood pressure, temperature, oxygen saturation), an ECG and basic blood parameters (small blood count, natrium, potassium and, if necessary, inflammation parameters, and D-dimer). Therefore, patients with an obvious hemiparesis or dyspnea were excluded, whereas patients with a subtle focal neurological deficit or mild internistic symptoms were included. In the ADVIUC analysis, all cases with symptoms lasting more than 72 h were excluded.

For all ADVIUC cases, discharge letters were requested, and relevant information was extracted by neurootologists in the stroke center (CL, PMB). Base data collected are as follows: age, sex, relevant risk factors, relevant symptoms, and hospital discharge diagnoses.

Accuracy of the triage algorithm was focused on cases with vestibular disorders. We compared the ED diagnoses to final discharge diagnoses for all central and peripheral vestibular cases according to the final discharge diagnosis. ED diagnoses were categorized as peripheral if diagnostic criteria for vestibular neuritis or BPPV were fulfilled. Cases with central signs (e.g., a direction-changing nystagmus or a skew deviation) or constellations suggestive for a central lesion (e.g., direction-fixed horizontal nystagmus, but physiological vHIT) or unclear constellations (e.g., no peripheral and central vestibular symptoms) were categorized as central.

In most ADVIUC cases without clear evidence for peripheral or internal medicine cause, a CT scan was performed in the ED. In all ADVIUC cases suspicious for a central etiology, cranial MRI was recommended between days 3 and 5. The written results of all brain scans performed were collected and evaluated by experienced vascular neurologists centrally (CL, PMB). For the evaluation of missed strokes, all ADVIUC cases with an adequate brain imaging were considered. All MRI scans were considered as adequate, whereas CT scans were counted only if pathologic. All cases in the spoke hospitals with CT or MRI evidence of stroke, which were not primarily admitted to stroke units, were counted as missed strokes.

For all acute telemedical ED triage examinations, subjective satisfaction and added value were documented by the teleconsultants. Accordingly, the same items were documented for all elective VOGs by the physicians who made the finding. For further project evaluation, an online questionnaire was sent to all participating hospitals at the end of the project phase in November 2020. We wrote to our medical contact persons and also to therapists and technicians with the request to forward the questionnaire to their colleagues. The questionnaire asked for the subjective assessment of how helpful measures within the project were perceived, whether the care of patients with dizziness in their own hospital was improved, whether their own competence could be expanded, how satisfactory the implementation of the VOG was perceived, and whether a continuation of the application was desired. Answers were given anonymously.

### Outcomes and Statistics

Primary outcome of this evaluation is the accuracy of the ED triage algorithm in differentiation between peripheral and central vestibular disease. Secondary outcomes are rate of missed strokes in acutely dizzy patients, improvement of diagnostic quality, and acceptance of physicians in spoke hospitals.

We applied descriptive statistics. Due to non-normal distribution of cases, data are presented as median and interquartile range. All patient data are derived from our anonymized and project-related quality register. According to German legislation, no patient consent is required for documentation in routine observational quality registries.

## Results

Screening lists were expected from all 19 hospitals in all of the three evaluation months (total *n* = 57) and provided from 18 hospitals in 40 evaluation months (40/57 = 70%). We had to exclude 8 lists due to implausible data, with 32 remaining lists from 15 hospitals (32/57 = 56%) and a total of 469 cases with the leading symptom of dizziness, vertigo, or imbalance in the ED. Response rate improved gradually over time from 6 to 12 to 14 hospitals with valid screening lists. The median number of cases was 13.5 per month and hospital [interquartile range (IQR) 10–21]. After exclusion of 81 cases with symptoms lasting more than 72 h, a total number of 388 ADVIUC cases remained with a median number of 12 per month and hospital (IQR 8–14.5; see Table 1).

**Table 1:** Number of cases per hospital and evaluation month with acute (<72 h from onset) dizziness, vertigo, or imbalance of unknown cause (ADVIUC) as a leading symptom for ED admission.

	**Month 1**	**Month 2**	**Month 3**	**Cases per year**
Agatharied	10	12	14	144
Bad Reichenhall	9	8	7	96
Bad Tölz	7	11	13	124
Burglengenfeld		8	12	120
Cham		12		144
Ebersberg		10	5	90
Eggenfelden			25	300
Erding			12	144
Freising		12	16	168
Mühldorf		7	12	114
Rosenheim	16	17	17	200
Rotthalmünster		12	6	108
Traunstein	18	20	24	248
Vilsbiburg			8	96
Wasserburg	13	9	6	112
Total	73	138	177	2,208
Median (IQR)		12 (8–14.5)		124 (110–184)

*Annual number of cases are extrapolated from monthly numbers*.

Baseline characteristics, risk factors, and symptoms of the 388 acute cases are given in Table 2. In 11.9% of cases, there is a history of dizziness or vertigo, but of different symptom quality. In most cases (87.9%), onset was reported as hyperacute. Nystagmus was detected in 51.0% of cases.

**Table 2:** Baseline characteristics of all cases with an acute leading symptom of dizziness, vertigo, or imbalance of unknown cause (ADVIUC).

	***n***	***N***	**%**
Age [median; IQR (years)]	65 (54–78)		
Female sex	210	388	54.1%
**Risk factors**			
Arterial hypertension	224	388	57.7%
Diabetes mellitus	68	388	17.5%
Hypercholesterinemia	146	388	37.6%
(ex-)smoker	59	388	15.2%
Atrial fibrilation	46	376	12.2%
Osteoporosis	17	359	4.7%
History of dizziness/vertigo	43	360	11.9%
**Symptoms**			
Hyperacute onset	340	387	87.9%
Dizziness or vertigo	386	388	99.5%
Vertigo only	206	370	55.7%
Imbalance	235	359	65.5%
Nystagmus	197	386	51.0%
Nausea	246	343	71.7%
Vomiting	153	340	45.0%
New neck pain or headache	49	263	18.6%
Known headache	22	388	5.7%
New tinnitus	21	214	9.8%
New hearing disturbance	14	211	6.6%
New ear symptoms (others)	20	211	9.5%
New phono- or photophobia	8	163	4.9%

*Missing cases are due to missing information in available hospital discharge letters*.

Distribution of hospital discharge diagnoses of these cases is displayed in Table 3 with vestibular neuritis, stroke/TIA and BPPV (each about 20%), and dizziness of internal medicine causes (15%) as the most frequent diagnoses.

**Table 3:** Distribution of hospital discharge diagnoses of all cases (*n* = 388) with an acute leading symptom of dizziness, vertigo, or imbalance of unknown cause (ADVIUC).

**Diagnosis**	***n***	**%**
Vestibular neuritis	87	22.4%
Vertebrobasilar stroke or TIA	68	17.5%
BPPV	68	17.5%
Dizziness of internal medicine cause	57	14.7%
Meniere's disease	26	6.7%
Functional disorder	7	1.8%
Vestibular migraine	7	1.8%
Inflammatory CNS disease	6	1.5%
Others^*^	3	0.8%
Unclear	59	15.2%

* *Others include one case of bilateral vestibulopathy, vestibular paroxysm, and polyneuropathy each*.

**Figure 4:**
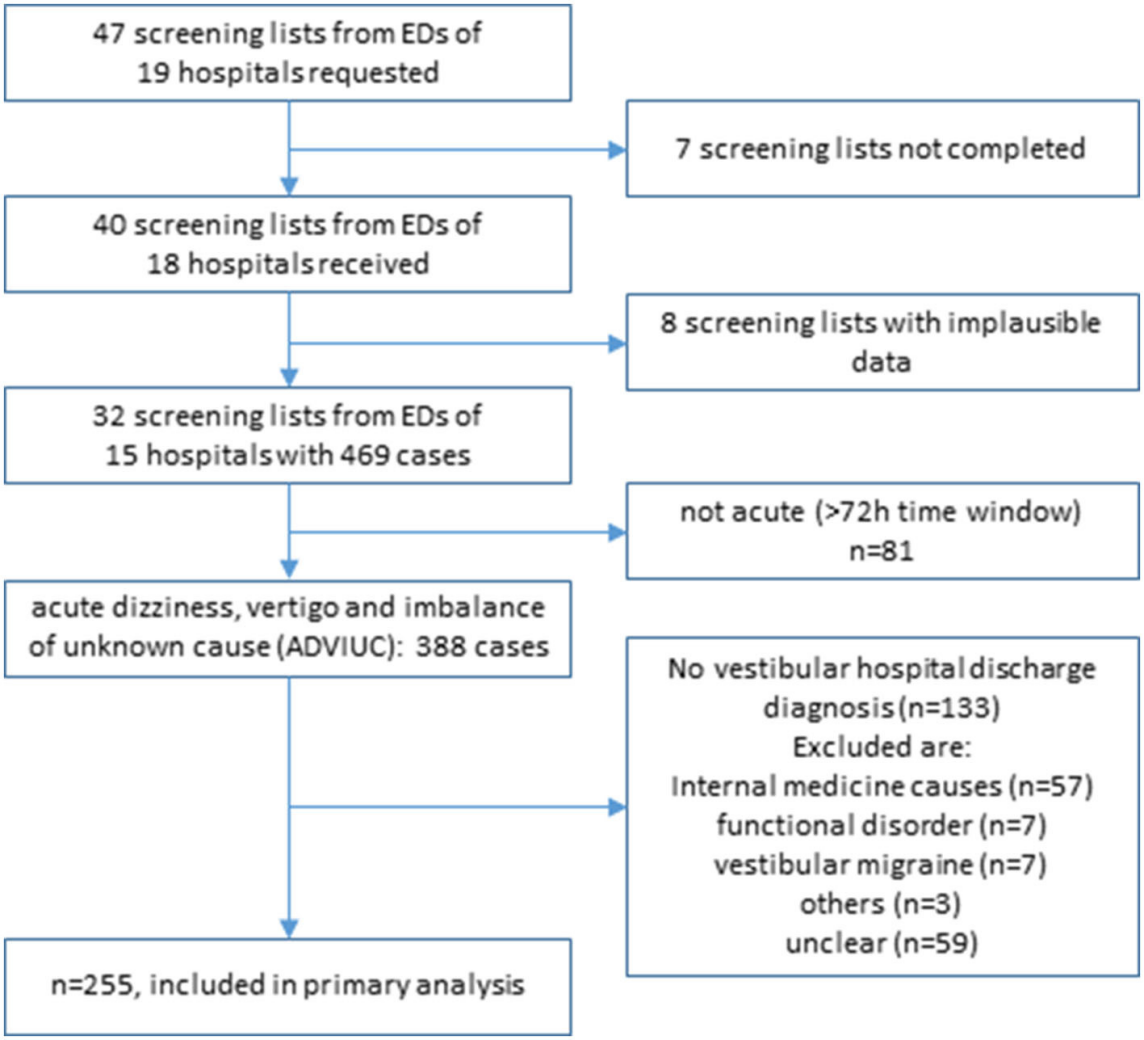
Flowchart of the study cohort.

Accuracy of the ED triage algorithm was calculated in vestibular cases compared to the final discharge diagnosis (see Figure 4). Sensitivity for detecting a central lesion was 73/74 (98.6%) and specificity 83/181 (45.9%). The positive predictive value is 73/171 (42.7%) and the negative predictive value 83/84 (98.8%) (see Table 4).

**Table 4:** Accuracy of ED triage algorithm in vestibular cases.

**ED diagnosis final diagnosis**	**Central vestibular**	**Peripheral vestibular**	**Total**
Central or unclear	73	98	171
Peripheral	1	83	84
Total	74	181	255
Sensitivity:	98.6%		
Specificity:	45.9%		
Positive predictive value:	42.7%		
Negative predictive value:	98.8%		

*ED diagnoses compared to final discharge diagnoses*.

In 196 cases, an adequate brain imaging (MRI in 192 cases and CT in 4 cases) was performed with a brain lesion matching the symptoms in 36 cases (36/196 = 18.4%). In four cases, lesions were not due to stroke: 1 acoustic neurinoma, 1 inflammatory lesion, 1 cerebral tumor, and 1 PICA aneurysm with compression of the brainstem. Three out of 32 stroke lesions were detected in CT scans (two ischemic cerebellar and one ischemic brainstem lesion), the remaining by MRI scans. A number of 16 out of 32 strokes were located predominantly in the cerebellum, 11/32 strokes in the brainstem, and 5/32 strokes in the cortex. One stroke was a cerebellar hemorrhage, the remaining ischemic. With the ED triage algorithm, 31/32 (96.9%) strokes confirmed by imaging were detected correctly in the ED. One stroke was missed (3.1%).

**Figure 5:**
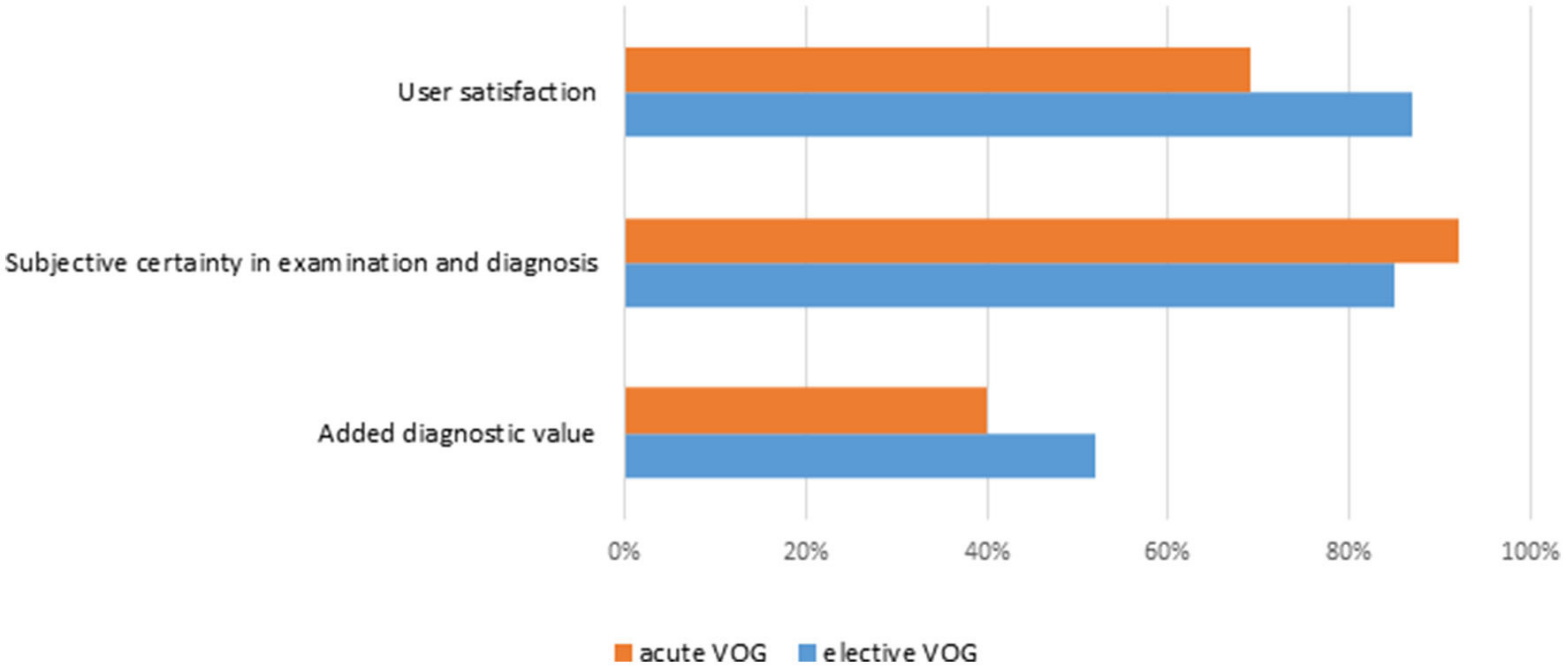
Subjective user assessment for acute and elective videooculographies. Rates of positive answers.

**Figure 6:**
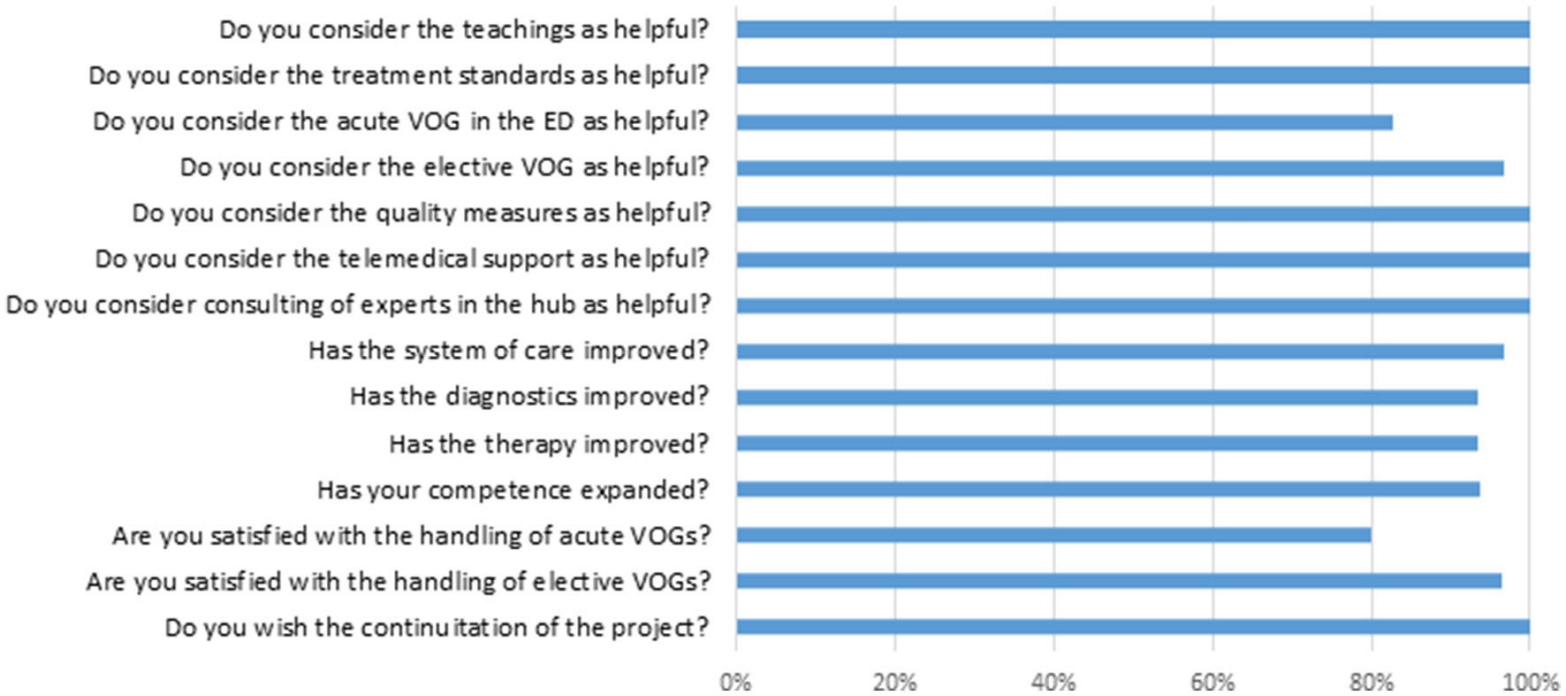
Results of online survey in November 2020. Rates of positive answers.

User satisfaction, the subjective sensation of safety in the clinical examination and diagnostic interpretation and the subjective added value in the concrete case by application of the acute and elective VOG are generally high, as stated by the teleconsultants and on-site physicians (see Figure 5). A total of 32 colleagues responded to our anonymous online questionnaire in November 2020. The total of 59% of them are physicians, 34% therapists and 6% technicians. Questions about helpfulness of the project, improvement of care on ADVIUC patients, and personal competence and also satisfaction about handling of the elective VOGs were rated consistently positive (for details see Figure 6). Slightly lower rates (80% positive) were recorded concerning helpfulness and satisfaction with the handling of acute VOGs. All responders voted for a continuation of the project.

## Discussion

We report on our experience of a telemedical approach to improve quality of care in patients with acute dizziness, vertigo, or imbalance in primary care hospitals. The approach is based on telestroke networks and comprises multiple components including the implementation of standards, training of ED physicians, on-site neurologists, on-site therapists and teleconsultants, telemedical examinations, and implementation of VOG including vHIT (see Figure 2). The implementation is challenging and needs relevant resources and time, but our data demonstrate its feasibility. The approach distinguishes central (mostly stroke) from peripheral vestibular disorders with high sensitivity, albeit poor specificity. The vast majority of colleagues involved report subjective satisfaction and added diagnostic value and also the desire to continue the project.

The median monthly number of ADVIUC cases in the EDs of our primary care hospitals is 12 (IQR 8 – 14.5). Our data allow us to estimate an incidence, because catchment areas of our primary care spoke hospitals correspond largely to the correlating county, as we know from earlier evaluations of stroke cases (30). An extrapolated annual number of 2,208 ADVIUC cases correspond to a population of 1,987,414 inhabitants, resulting in an annual incidence of 111 cases per 100,000 inhabitants. This is in line with population-based studies performed by retrospective medical record reviews. A study from Sweden (4) reports an incidence of acute vestibular syndrome in the ED of 92 per 100,000 inhabitants per year, and another from Texas (6), reports 1,297 isolated dizziness symptoms as the principal presenting complaint in a 3.5-year period in proportion to about 300,000 inhabitants, and therefore resulting in an incidence of 123.5 cases per 100,000 inhabitants per year.

Our ADVIUC cases in ED of primary care hospitals resulted in the final diagnosis of vestibular neuritis, stroke/TIA and BPPV in about 20% of cases each, and dizziness of internal medicine cause in about 15%. These figures differ relevantly from other publications. Ljungren report 10% stroke/TIA in their sample of acute vertigo in the ED (4). However, it must be mentioned that only 45% of cases got a medical diagnose, whereas 55% were coded with a symptom diagnose. In an US sample of dizziness presentations in the ED, 4% resulted in a diagnosis of stroke/TIA, 33% otologic/vestibular, 21% cardiovascular, and 11% respiratory and metabolic (5). Noteworthy is again that only 49% of cases got a medical diagnose and the sample was not restricted to acute cases without an obvious underlying reason for the chief complaint of dizziness. Therefore, we believe that differences are explained by a selection bias due to different sampling definitions and an additional coding bias (in our sample in 15% of cases the final diagnosis remained unclear). Data for acute vertigo/dizziness from the ED of a tertiary referral center seem to match our data quite well-considering that recurrent manifestations of episodic syndromes are not excluded and medical disorders may be included more frequently (31).

The high proportion of 20% of stroke/TIA diagnoses in our sample underlines the importance for rapid and accurate triage of dizzy patients in the ED. Our approach (Figure 3) is highly sensitive (98.6%), but of poor specificity (45.9%). Only one stroke out of 32 verified by brain scan was missed, which outperforms misdiagnosis rates of 35% and more reported earlier (6–9). One reason is our conservative approach in unclear cases in the ED: until a peripheral cause is not proven, a central cause is assumed. Therefore, two-thirds of the cases were primarily admitted to stroke units (171/255 cases). Retrospectively, this was unnecessary in 57% of cases (98/171). Further improvement in triage tools is mandatory to reduce overadmission to stroke unit.

During planning and implementation of our TeleVertigo project, a strong feeling for the need of a specific concept for the management of dizzy patients in the ED of primary care hospitals evolved. Spectrum of diagnoses, training and experience of ED physicians and on-site neurologists, and diagnostic resources differ relevantly from other settings like at the general practitioner, in outpatient clinics, and in academic and specialized vertigo centers. Therefore, data and approaches cannot be transferred easily between these settings. In the literature, we could not find a commonly accepted definition for the relevant symptoms in our ED setting. There are various and substantially different definitions for acute vestibular syndrome (AVS) (4, 5, 12, 32), mostly requiring a persistence of symptoms over at least 24 h and a nystagmus. There is not an accepted definition for acute vertigo. Furthermore, vertigo and dizziness are different symptoms by definition (29), but cannot be distinguished by anamnesis in the ED in a relevant percentage of cases (33, 34). Therefore, we suggest a new definition for use in the ED setting, which focuses on the diagnostically challenging cases. These are all patients with acute dizziness, vertigo, or imbalance of unknown cause (ADVIUC). We defined “acute” as symptoms persisting <72 h. The reason for this is the consideration that for strokes with onset > 72 h stroke unit treatment usually is no longer indicated and therefore rapid triage to stroke unit or normal ward is unnecessary. “Unknown cause” means without an obvious reason for these symptoms detectable in the first medical check by ED physicians at admission (like an obvious focal neurological deficit, acute respiratory distress or relevant tachycardia). This excludes also recurrent manifestations of an already diagnosed episodic disorder, as long as the acute symptoms are preknown. In our cohort of ADVIUC cases in the ED, any nystagmus was detected in 51% of cases only. Cases without nystagmus (*n* = 189) correspond mostly to cases with dizziness of internal medicine cause (52/189), stroke/TIA (42/189), and unclear cases (44/189) and emphasize the difference between AVS and ADVIUC.

We believe that for these ADVIUC patients in this setting, a comprehensive approach for adequate diagnostic and therapy is required. The diagnostic approaches based on the HINTS rule alone do not seem sufficient and feasible. Diagnostic accuracy has only been shown in highly selected patients due to tight inclusion criteria (35). The HINTS rule does not work at all in cases with completely resolved symptoms such as TIA or BPPV. The generalizability of the positive study results to less selective cohorts appears problematic. Furthermore, in our experience, an independent application and interpretation of the HINTS rule by ED physicians in primary care hospitals rarely yields reliable results. Nevertheless, HINTS is a precious diagnostic tool and is implemented in our ED triage algorithm as well (see Figure 3). Essentially, it is embedded in a telemedically supported and network-based application to assure specific feasibility. Moreover, questions about internal medicine cause and Dix-Hallpike maneuvers are added, and the workflow is optimized. The teachings and trainings for teleconsultants, ED-physicians, on-site neurologists, technicians, and physiotherapists are focused on history-taking [especially about timing and triggers according to the TiTrATE concept (36, 37)], the examination of subtle focal neurological symptoms and central oculomotor signs and also the four major diagnoses involved (vertigo/dizziness of central cause, vestibular neuritis, BPPV and, internal medicine causes of dizziness). We assume that this knowledge is important to understand diagnostic and treatment standards and therefore improves adherence to standards and quality of care.

One strength of this study is the prospective data sampling with active screening of all ED cases. Consequently, we can report reliable numbers of cases of difficult dizziness presentations in the ED of primary care hospitals and also a reliable diagnostic spectrum. Hospital discharge diagnoses in the spokes were made thoroughly based on the established treatment standards and under supervision of the network center. Accuracy of the ED triage algorithm was prospectively analyzed.

Some limitations have to be addressed. First, although hospital discharge diagnoses in the spokes were made in the best way possible, the diagnostic quality does not reach standards of specialized academic centers and leaves room for misdiagnoses. MRI scans were only conducted on cases with suspicion for a central etiology; therefore, some central lesions may have been missed. Furthermore, analysis of accuracy of the ED triage was restricted to cases with a vestibular final discharge diagnosis, as the major challenge in the ED is the correct identification of stroke and TIA cases in ongoing but also resolved dizziness symptoms. Finally, we did not verify the proficiency of all staff members involved and did not systematically survey the quality of VOG examinations and diagnoses in the spoke hospitals. This system is aimed to improve quality of care in a large area with a clearly defined protocol to serve as many patients as possible. This expansion of competence into the area may be at the expense of the competence of the individual actors – a conflict you often face when setting up large scale systems of care. In diagnoses with potentially time dependent treatment (such as acute stroke), this strategy has been proven to be effective (18). The overall system, which includes the protocol and proficiency of all staff involved, seems very reassuring regarding the most important aim, the detection of an acute central cause.

In conclusion, the concept evaluated shows good acceptance for a complex telemedical and network-based approach to manage patients with acute dizziness, vertigo, or imbalance of unknown cause in the ED of primary care hospitals. Identification of stroke cases is accurate, whereas specificity needs further improvement. The concept could be a major step toward a broadly available evidence-based and state of the art diagnostics and therapy for patients with ADVIUC, especially in primary care hospitals of rural areas.

## Data Availability Statement

The raw data supporting the conclusions of this article will be made available by the authors, without undue reservation.

## Ethics Statement

Ethical review and approval was not required for the study on human participants in accordance with the local legislation and institutional requirements. Written informed consent for participation was not required for this study in accordance with the national legislation and the institutional requirements.

## Author Contributions

PM-B, CL, NH, and GH contributed to conception and design of the study. CL and PM-B organized the database and performed the statistical analysis. PM-B wrote the first draft of the manuscript. All authors contributed to manuscript revision, read, and approved the submitted version.

## Funding

NS-B, CL, JP, and PM-B efforts were supported by a grant from the Bavarian Ministry of Health and the German Foundation for Neurology (DSN).

## Conflict of Interest

HR is beta-tester of the Otosuite® VOG system but has no financial interest in the product. He received honorary from Natus Medical ApS, Denmark, and Henning-Arzneimittel, Germany. PM-B, CL, JP, and NS-B report grants from the Bavarian Ministry of Health and the German Foundation for Neurology DSN during the conduct of the study. The remaining authors declare that the research was conducted in the absence of any commercial or financial relationships that could be construed as a potential conflict of interest.

## Publisher's Note

All claims expressed in this article are solely those of the authors and do not necessarily represent those of their affiliated organizations, or those of the publisher, the editors and the reviewers. Any product that may be evaluated in this article, or claim that may be made by its manufacturer, is not guaranteed or endorsed by the publisher.

## Abbreviations

ADVIUC, acute dizziness, vertigo: or imbalance of unknown cause
BPPV: benign paroxysmal positional vertigo
HINTS: head impulse &#8211
nystagmus &#8211
test of skew
IQR: interquartile range
TEMPiS: Telemedical Project for Integrative Stroke Care
TiTrATE, timing, triggers: and targeted bedside eye examinations
TIA: transient ischemic attack
vHIT: video head impulse test
VOG: videooculography.
